# Assessing the Prevalence and Potential Risks of *Salmonella* Infection Associated with Fresh Salad Vegetable Consumption in the United Arab Emirates

**DOI:** 10.3390/foods12163060

**Published:** 2023-08-15

**Authors:** Ihab Habib, Mushtaq Khan, Mohamed-Yousif Ibrahim Mohamed, Akela Ghazawi, Afra Abdalla, Glindya Lakshmi, Mohammed Elbediwi, Hassan Mohamed Al Marzooqi, Hanan Sobhy Afifi, Mohamed Gamal Shehata, Rami Al-Rifai

**Affiliations:** 1 Veterinary Public Health Research Laboratory, Department of Veterinary Medicine, College of Agriculture and Veterinary Medicine, United Arab Emirates University, Al Ain P.O. Box 1555, United Arab Emirates; mohamed-yousif-i@uaeu.ac.ae (M.-Y.I.M.); afra.abdalla@uaeu.ac.ae (A.A.); glindya_l@uaeu.ac.ae (G.L.); 2 Environmental Health Department, High Institute of Public Health, Alexandria University, Alexandria 21531, Egypt; 3 Department of Medical Microbiology and Immunology, College of Medicine and Health Sciences, United Arab Emirates University, Al Ain P.O. Box 1555, United Arab Emirates; mushtaq.khan@uaeu.ac.ae (M.K.); akelag@uaeu.ac.ae (A.G.); 4 Zayed Center for Health Sciences, United Arab Emirates University, Al Ain P.O. Box 15551, United Arab Emirates; rrifai@uaeu.ac.ae; 5 Evolutionary Biology, Institute for Biology, Freie Universität Berlin, 14163 Berlin, Germany; mohammed.elbediwi@fu-berlin.de; 6 Animal Health Research Institute, Agriculture Research Centre, Cairo 12618, Egypt; 7 Food Research Section, Research and Development Division, Abu Dhabi Agriculture and Food Safety Authority (ADAFSA), Abu Dhabi P.O. Box 52150, United Arab Emirates; hassan.marzouqi@adafsa.gov.ae (H.M.A.M.); hanan.afifi@adafsa.gov.ae (H.S.A.); mohamed.shehata@adafsa.gov.ae (M.G.S.); 8 Food Technology Department, Arid Lands Cultivation Research Institute (ALCRI), City of Scientific Research and Technological Applications (SRTACITY), New Borg El-Arab City 21934, Egypt; 9 Institute of Public Health, College of Medicine and Health Sciences, United Arab Emirates University, Al Ain P.O. Box 1555, United Arab Emirates

**Keywords:** *Salmonella*, WGS, leafy greens, Middle East, risk analysis

## Abstract

This study aimed to investigate the occurrence and characteristics of *Salmonella* isolates in salad vegetables in the United Arab Emirates (UAE). Out of 400 samples tested from retail, only 1.25% (95% confidence interval, 0.41–2.89) were found to be positive for *Salmonella*, all of which were from conventional local produce, presented at ambient temperature, and featured as loose items. The five *Salmonella*-positive samples were arugula (n = 3), dill (n = 1), and spinach (n = 1). The *Salmonella* isolates from the five samples were found to be pan-susceptible to a panel of 12 antimicrobials tested using a disc diffusion assay. Based on whole-genome sequencing (WGS) analysis, only two antimicrobial resistance genes were detected—one conferring resistance to aminoglycosides (*aac(6′)-Iaa*) and the other to fosfomycin (*fosA7*). WGS enabled the analysis of virulence determinants of the recovered *Salmonella* isolates from salad vegetables, revealing a range from 152 to 165 genes, collectively grouped under five categories, including secretion system, fimbrial adherence determinants, macrophage-inducible genes, magnesium uptake, and non-fimbrial adherence determinants. All isolates were found to possess genes associated with the type III secretion system (TTSS), encoded by *Salmonella* pathogenicity island-1 (SPI-1), but various genes associated with the second type III secretion system (TTSS-2), encoded by SPI-2, were absent in all isolates. Combining the mean prevalence of *Salmonella* with information regarding consumption in the UAE, an exposure of 0.0131 salmonellae consumed per person per day through transmission via salad vegetables was calculated. This exposure was used as an input in a beta-Poisson dose–response model, which estimated that there would be 10,584 cases of the *Salmonella* infection annually for the entire UAE population. In conclusion, salad vegetables sold in the UAE are generally safe for consumption regarding *Salmonella* occurrence, but occasional contamination is possible. The results of this study may be used for the future development of risk-based food safety surveillance systems in the UAE and to elaborate on the importance for producers, retailers, and consumers to follow good hygiene practices, particularly for raw food items such as leafy salad greens.

## 1. Introduction

Fresh salad vegetables have become a regular component of our daily dietary intake due to their high nutritional value, delicate texture, and pleasing taste [1]. Unfortunately, fresh salad vegetables have been linked to food recalls and outbreaks of foodborne illnesses due to the possibility of contamination with pathogens during several stages of their lifecycle, including production, harvesting, post-harvest handling, packaging, distribution, and retail [2,3]. When consumed, bacterial pathogens present in these vegetables can potentially result in foodborne illnesses [4].

Based on epidemiological investigations, it has been revealed that non-typhoidal *Salmonella* is among the frequent bacterial pathogens responsible for foodborne outbreaks associated with fresh salad vegetables [5,6]. The prevalent hypothesis is that consuming raw salad vegetables poses a risk of salmonellosis due to cross-contamination arising from the preparation of food with other contaminated items, such as raw chicken [7]. However, numerous studies have reported the detection of *Salmonella* on raw fresh produce at the retail level before food preparation [8,9,10]. A meta-analysis conducted on non-typhoidal *Salmonella* in vegetables (packed and unpacked) indicated that leafy salad greens had a higher prevalence of this pathogen than other categories under scrutiny, with bulk/unpacked vegetables consistently exhibiting a higher pooled prevalence than packed vegetables [11]. Non-typhoidal *Salmonella*-induced gastroenteritis is typically a self-limiting illness characterized by fever, diarrhea, vomiting, and abdominal pain. Nevertheless, individuals such as children, the immunocompromised, and the elderly are at higher risk of developing the severe disease with a greater likelihood of secondary complications [12].

In the past decade, fresh leafy greens in the United Arab Emirates (UAE) have seen a rise in sourcing from soil-less farms that are operated locally and utilize vertical facilities; aeroponic and hydroponic-based cultivation methods to improve water efficiency are crucial in the harsh arid climate of the UAE [13]. The local harvest accounts for approximately 15% of the fresh vegetable supply chain in the UAE, while the majority of the national demand for salad vegetable items is met by importing them from various regional and international markets [13]. Research on the hygiene and safety of plant-based food in the UAE is scarce, especially compared to animal-based food [9]. No published evidence exists regarding the status of non-typhoidal *Salmonella* in leafy greens sold in the UAE, which hinders the development of a reliable risk assessment of the burden of this critical pathogen in one of the most frequently consumed raw food categories in the UAE’s daily diet.

Quantitative risk assessments facilitate the numerical evaluation of the potential health hazards arising from pathogens in our food chain. Risk assessment outcomes can be employed to devise scientifically based strategies for managing the associated risks and protecting public health [14]. Due to the prevalent consumption of fresh salad vegetables in their raw state, without an explicit cooking process before consumption, developing risk assessment models targeting these products is of utmost importance [15]. The primary objective of this study is to generate the first baseline data on the occurrence of non-typhoidal *Salmonella* in salad vegetables presented at retail in the UAE, based on an extensive array of samples. Additionally, the phenotypic antimicrobial resistance features of the *Salmonella* strains recovered were characterized, and their serotypes and other genome-based attributes were deduced using whole-genome sequencing. Moreover, a simplified risk assessment was performed to estimate the likelihood of infection resulting from human exposure to *Salmonella* by consuming salad vegetables in the UAE.

## 2. Materials and Methods

### 2.1. Baseline Survey and Study Setting

In order to account for all potential routes of contamination, a sampling frame was established by acquiring samples of fresh salad vegetables from retail locations. This sampling frame included samples from public vegetable markets (n = 173) and supermarket chains (n = 227) in the cities of Abu Dhabi, Al Ain, and Dubai to obtain an adequate representation of domestic (n = 290) and imported (n = 110) products and those from conventional (n = 321) and soil-less cultivates (n = 79). The latter has gained significant popularity in the UAE agriculture sector in recent years and involves the indoor cultivation of crops using controlled climate and lighting, and are typically operated as vertical farming sites [13].

The sample number was determined based on a prevalence rate of 50%, a confidence level of 95%, and 5% error margin [16]. Four hundred samples were collected and analyzed from May 2022 to January 2023, over ten months. The samples included the following items; arugula (n = 51), dill (n = 18), spinach (n = 49), cabbage (n = 36), coriander (n = 31), iceberg lettuce (n = 31), roman lettuce (n = 51), lettuce-others varieties (n = 48), onion leaves (n = 15), parsley (n = 39), and mixed packs (n = 31). During the collection process, the collected items were assessed for the absence of visible dirt and spoilage. To ensure the preservation of the samples’ condition, they were kept in a cooling box, firmly sealed with a sterile wrap, and subjected to laboratory testing within the same day of collection.

### 2.2. Salmonella Detection and Confirmation

Each tested sub-sample weighed 25 g and was thoroughly chopped using sterile stainless-steel scissors. The resulting material was homogenized with 225 mL of buffered peptone water (BPW; Oxoid, UK) for a duration of 2 min using a BagMixer (Interscience, Saint Nom la Brétèche, France). *Salmonella* was detected in the sample homogenate following the guidelines outlined in the International Organization for Standardization ISO 6579-1:2017 [17]. In brief, the sample homogenate was incubated overnight at 37 °C; subsequently, a 100 µL aliquot of the pre-enriched suspension was transferred to 10 mL of Rappaport-Vassiliadis broth (RV; Oxoid, UK) and incubated at 42 °C for 24 h. A loopful of the broth culture was then streaked onto Xylose Lysine Deoxycholate (XLD; Oxoid, UK) agar, followed by incubation at 37 °C for 24 h [17].

The presence of suspected *Salmonella* was identified based on the characteristic colony morphology. The confirmation of suspected colonies was carried out at the genus level using a real-time PCR kit (*Salmonella*_*invA*-EASY genesig^®^ Kit; PrimerDesign Ltd., Eastleigh, UK), which detects the invasion protein (*InvA*) gene of *S. enterica* [18].

### 2.3. Whole-Genome Sequencing (WGS)-Based Characterization

In order to enhance the characterization of the confirmed *Salmonella* isolates, WGS was employed; for this purpose, the DNA was isolated utilizing a commercially available kit (Wizard^®^ Genomic DNA Purification Kit, Promega, Madison, WI, USA). The WGS was performed on the Illumina NovaSeq platform PE150 (Illumina, San Diego, CA, USA), which was carried out by a commercial send-out service provider, Novogene, based in the United Kingdom. The process of preparing the library involved using an Illumina NexTera^®^ XT kit. Bioinformatics techniques were employed to analyze the data obtained from WGS, including computing serovars, multi-locus sequence types, and antimicrobial resistance genes. The analyses were carried out as described in our previous work [18], and the genomic sequences were aligned in the Virulence Factor Database (VFDB) database (http://www.mgc.ac.cn/VFs/; accessed on 15 February 2023) to obtain annotations of the virulence function. The assemblies of *Salmonella* genomes were searched against VFDB using BLAST with an identity of genes defined at ≥90% and a cut-off value of 50% of the sequence length [19].

All of the generated genome data in the present work have been submitted in the National Center for Biotechnology Information (NCBI) Sequence Read Archive under bioproject accession number PRJNA981417.

### 2.4. Antimicrobial Susceptibility Testing

The study used breakpoint values established by the Clinical and Laboratory Standards Institute to assess the antimicrobial susceptibility phenotypes of the confirmed *Salmonella* isolates [20]. The disc diffusion method was used to evaluate antibiotics, including ampicillin, azithromycin, ciprofloxacin, tetracycline, gentamicin, chloramphenicol, trimethoprim–sulfamethoxazole, cefoxitin, cefotaxime, ceftriaxone, cefepime, and imipenem [18]. The strain *E. coli* ATCC 25922 was included for a quality check in each testing run. The strains were denoted as multi-drug resistant (MDR) if they were resistant to at least one agent in three or more antimicrobial classes using breakpoints set by the CLSI [21].

### 2.5. Risk Assessment Model Development

The input data on the prevalence of non-typhoidal *Salmonella* in salad vegetables at retail (*P_ret_*) was based on the results obtained from the current baseline study (Table 1), and the variability in the prevalence was simulated in the model by a beta distribution (Table 2). The Emirate of Abu Dhabi report’s Food Balance Sheet Survey (for the year 2019) was used to surrogate an input variable describing the average daily consumption of raw salad vegetables in the UAE [22]. According to that report, the average amount of raw salad vegetables consumed daily per individual (*M_veg_*) was 23.6 g—this was rounded to 24 g. To account for variability in consumption (serving size), the *M_veg_* was modeled using Pert distribution parameters of 12 g, 24 g, and 36 g, assuming a hypothetical minimum, mode, and maximum serving sizes, respectively [23] (Table 2).

No enumeration data were generated in this study; however, the detection of *Salmonella* spp. was determined in 25 g of the tested salad vegetables. The minimal concentration (*C*) of *Salmonella* in the contaminated samples was substituted in the calculations, which is equivalent to a Poisson distribution of the limit of detection (1 CFU/25 g) [15] (Table 2). This approach was acquired based on a Dutch study assessing *Campylobacter* infection risk from consuming raw vegetables and fruits [15]. The average exposure (*D*), expressed as the average number (exact number, and not on a log scale) of *Salmonella* bacteria ingested at the UAE population level per person per day via a serving of salad vegetables, was modeled as a Poisson distribution of the multiplication of the prevalence of *Salmonella* (*P_ret_*), average daily consumption (*M_veg_*) per individual in the UAE, and *Salmonella* concentration (*C*) in per gram of salmonella-positive samples [15] (Table 2).

The calculated exposure (*D*) was used to estimate the probability of infection with *Salmonella* per day (*P_inf,day_*), using a beta-Poisson dose–response formula. The model’s parameters and values were drawn based on the risk assessment of *Salmonella* in poultry products, representing a commonly accepted dose–response formula used previously in the risk assessments of *Salmonella* in leafy greens [7,24]. Then, the probability of infection per individual per year (*P_inf,year_*) was calculated as *P_inf,day_* × 365. The number of expected cases of infection around the year for the UAE population *Cs_UAE,year_* then equals *P_inf,year_* multiplied by the number of inhabitants (=10.2 × 10^6^, based on the Worldometer elaboration [25] (Table 2)).

The @Risk software version 8.3.2 (Palisade Corporation, Newfield, New York, NY, USA) was used to run the model (Monte Carlo simulation) that was built in an Excel spreadsheet. The model estimated the probability of infection per individual per year (*P_inf,year_*) and the expected number of infection cases per year (*Cs_UAE,year_*), with 100,000 iterations being performed to generate these estimates [26]. For each iteration, the model estimated the probability of infection based on a random scenario of consumer exposure to *Salmonella* in a contaminated serving. Using the Spearman rank correlation coefficient (*r*), the analysis of sensitivity was conducted to determine the impact of input variables on the model output (the probability of infection with *Salmonella* per serving per day [*P_inf,day_*]).

## 3. Results and Discussion

### 3.1. Salmonella Prevalence in the UAE Salad Vegetables

In the present study, out of 400 samples tested from retail sources, only 5 samples (1.25% [95% confidence interval, 0.41–2.89]) tested positive for *Salmonella* (Table 1). All of the five samples were from conventional local produce (none were from imported samples nor soil-less harvest), were featured as loose items (not packaged/wrapped), and were presented at an ambient temperature (unrefrigerated). The five *Salmonella*-positive samples were arugula (3/51), dill (1/18), and spinach (1/49) (Table 1).

According to our findings, all salad vegetable samples that tested positive for *Salmonella* at retail outlets in the UAE were leafy vegetables (arugula, spinach, and dill). This result is aligned with previous studies revealing that leafy greens have a greater tendency for surface attachment, which makes the chances of *Salmonella* survival higher [2]. The positive samples were all loose items and were not refrigerated. This display method may contribute to the proliferation of *Salmonella* and other bacteria at the point of sale. Vendors were observed spraying water on fresh leafy salad items to maintain their freshness for longer periods, which could potentially introduce pathogens to the product if contaminated water is used. Additionally, this practice can facilitate the transfer of microbes across the cut surfaces of the produce [27].

The current study’s isolation rates for *Salmonella* are relatively lower than those reported in Malaysia, where coriander and lettuce salads had 52% and 32% isolation rates, respectively [28]. However, the current rates are higher than those reported in the United States, with *Salmonella* detection rates of only 0.06% and 0.34% for lettuce and coriander, respectively [29]. Furthermore, no *Salmonella* was found in raw vegetable samples from various European Union countries, Canada, and Australia [11,30].

According to the results in Table 1, the five contaminated samples in the present study were harboring four different *Salmonella* serovars and multi-locus sequence types (28:z4,z24:z6 (novel ST), Meleagridis (ST463), Mbandaka (ST3760), Anatum (ST64)). Despite the low prevalence, such a finding indicates the diversity of *Salmonella* types contaminating raw vegetables in the UAE. Fresh-vegetable-related disease outbreaks have recorded various produce items and *Salmonella* serovars, including those detected in the present study [1,5]. Alfalfa sprouts were found to be responsible for outbreaks of *S*. *meleagridis* in the United States of America, according to Sivapalasingam et al. [31]. Similarly, *S*. *mbandaka* was associated with outbreaks connected to alfalfa sprouts and sesame seeds in the United States of America [32] and Serbia [33], respectively. *S*. *anatum* has also been linked to foodborne outbreaks, caused by other food types [34]. Therefore, detecting these serovars in raw vegetables in this study is a cause for concern, mainly since raw salad vegetables are frequently consumed by most individuals in the UAE and worldwide daily. Preventing foodborne bacteria can be achieved by proper management during the pre-harvest stage and by implementing better post-harvest practices throughout the handling, distribution, and storage processes, which are crucial for ensuring the safety of fresh salad vegetables. This can include actions such as washing, using disinfectants, and peeling the vegetables before consumption [27].

### 3.2. Phenotypic and Genotypic Resistance to Antimicrobials

A susceptibility test against a panel of 12 antimicrobials was performed using disc diffusion. The results were categorized into three classes: resistant, intermediate, or susceptible. All five *Salmonella* isolates were susceptible to all 12 antimicrobials (pan-susceptible to the tested panel) (Table 1). As presented in Table 1, only two antimicrobial resistance genes were detected, with one known to confer resistance to aminoglycosides (*aac(6′)-Iaa*) and was presented in all isolates. The other resistance gene was the one conferring non-susceptibility to fosfomycin (*fosA7*), which was present in one isolate (*S*. *meleagridis*-ST463, isolated from a dill sample).

Whole-genome sequencing helped food safety research to reliably predict the antimicrobial characteristics in various foodborne bacteria, including *Salmonella* [18,19]. Based on our study results, the resistance profiles of the examined isolate, both phenotypic and genotypic, suggest that fresh salad vegetables may be a limited venue of human exposure to antimicrobial-resistant *Salmonella* in the UAE. Despite the presence of the *aac(6′)-Iaa* gene in the genome of all *Salmonella* isolates, there was no association with phenotypic resistance. Studies have shown that this gene is often weakly expressed or not expressed, resulting in aminoglycoside resistance in these strains as a consequence of regulatory mutations causing an increase in the gene expression [35]. On the other hand, our findings reveal the first detection of the *fosA7* gene in *S*. *meleagridis* obtained from the food chain in the UAE. Initially identified on the chromosomes of *Salmonella* Heidelberg isolated from broilers in Canada in 2017, *fosA7* has since been detected across different countries in various *Salmonella* serotypes (e.g., Derby, Agona, and Brandenburg) from various sources [36]. The chromosomally borne *fosA7* can enable bacteria to survive under low levels of fosfomycin and has the potential to serve as a recipient of the Fosfomycin-mobilized resistance gene as it can induce high-level resistance to fosfomycin and enhance bacterial fitness in *Salmonella* if the gene is instead transferred on plasmids [37]. Hence, detecting this gene in *Salmonella* from food typically consumed raw (dill) (Table 1), as found in our study, is worrisome.

### 3.3. Genome Insight on Virulence Determinants

**Figure 1:**
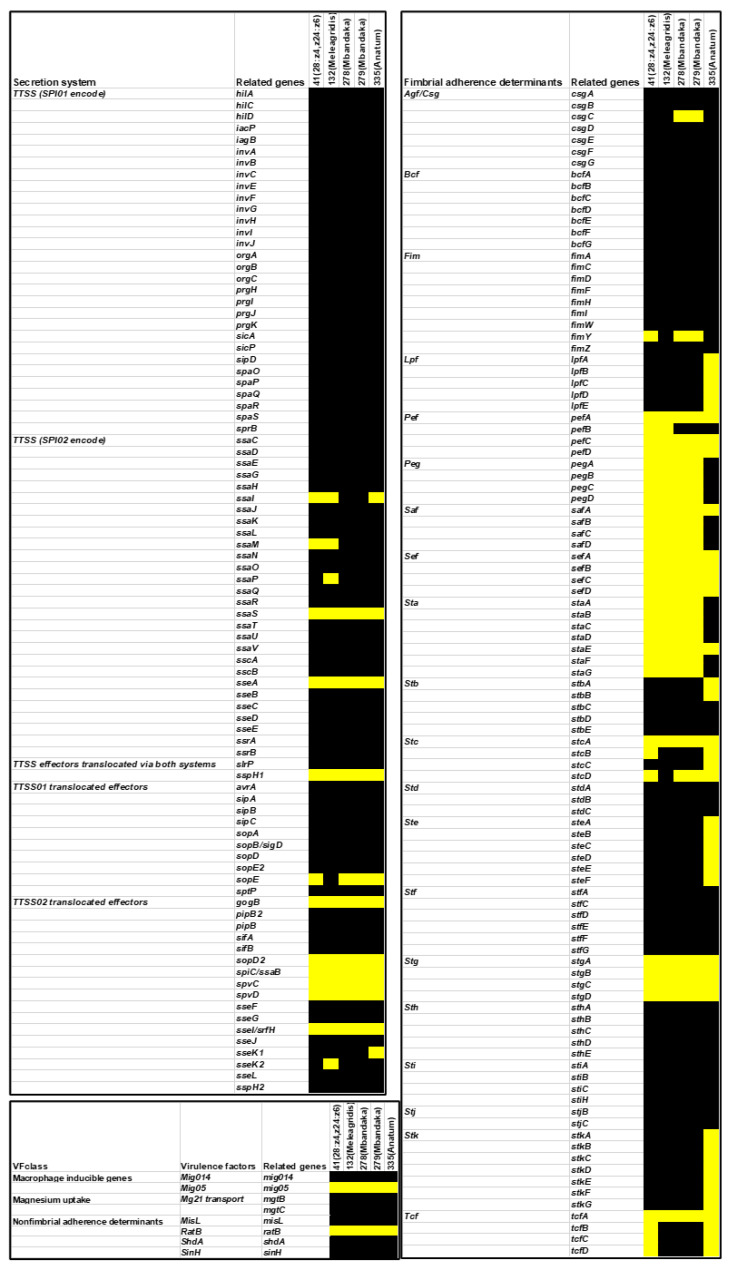
The presence (black) and absence (yellow) of virulence genes in *Salmonella* isolated from salad vegetables sampled at retail in the United Arab Emirates. Each row represents a virulence gene, and each column denotes a sample. The figure denotes the following categories of virulence genes: (I) secretion system, (II) fimbrial adherence determinants, (III) macrophage-inducible genes, (IV) magnesium uptake, (V) non-fimbrial adherence determinants.

The virulence-associated genes among *Salmonella* isolates from salad vegetables in the UAE were analyzed using whole-genome sequencing (Figure 1). The virulence genes ranged from 152 genes in isolate 335 (*S*. *anatum*) to 165 genes in isolates 278 and 279 (*S*. *mbandaka*). The virulence-associated genes were assigned into five categories (Figure 1): secretion system, fimbrial adherence determinants, macrophage-inducible genes, magnesium uptake, and non-fimbrial adherence determinants.

All isolates were found to possess the genes associated with the type III secretion system (TTSS) that are encoded by the *Salmonella* pathogenicity island-1 (SPI-1). However, only two genes, *ssaS and sseA*, which are necessary for the translocation of effectors from *Salmonella* pathogenicity island-2 (SPI-2) into host cells [1,19], were absent from all five isolates (Figure 1). Moreover, various genes (*gogB*, *sopD2*, *spiC/ssaB*, *spvC/D*, *sseL*, *srfH*) that are associated with the second type III secretion system (TTSS-2) encoded by SPI-2 were also absent in all isolates (Figure 1). These genes are involved in the translocation of proteins across the vacuolar membrane, which act collectively to enable intracellular replication—an essential step for *Salmonella* systemic infection [19].

The genes belonging to the *csg* operons, which encode for curli fimbriae and facilitate binding to various serum and tissue matrix proteins [38], were universally present in all isolates, except for the *csgC* gene, which was absent in two isolates (Figure 1). The gene clusters encoding for *Bcf* fimbrial genes and the *fim* genes, encoding for type 1 fimbriae, were also universally present. However, the *fimY* gene was not evident in three isolates at the BLAST search cut-off level used. Conversely, the *sef*, *stg*, *pef*, *sta*, and *stc* fimbrial operons gene clusters were the least-presented virulence genes and were infrequently detected across the *Salmonella* isolates characterized in this study (Figure 1).

Our findings indicate that Salmonella isolated from salad vegetables potentially causes human disease, as demonstrated by the universal presence of fimbrial genes and pathogenicity island 1 and 2 genes in these isolates [19]. The utilization of WGS allows for a thorough investigation of bacterial virulence genes and further enhances our understanding of the pathogenicity of these isolates [18,19]. As such, the genomes we presented in this work could stand as a reference for future local investigations, particularly in incidences where leafy greens might be suspected for human salmonellosis in the UAE.

### 3.4. Infection Risk with Salmonella Due to the Consumption of Salad Vegetables in the UAE

Adjoining the mean prevalence revealed in this survey with information regarding consumption in the UAE (refer to Table 2), we calculated the exposure of 0.0131 salmonellae consumed per individual a day through transmission via salad vegetables (Table 3). This exposure was used as an input in a beta-Poisson dose–response formula, which estimated that there would be 10,584 cases of *Salmonella* infection annually for the entire UAE population (Table 3). The sensitivity analysis in the present model identified uncertainty in the counts of *Salmonella* to be the most uncertain determinant of the probability of infection per serving per day (estimated as 2.367 × 10^−5^ (Table 3)).

The use of a quantitative risk assessment is an effective means of analyzing intricate exposure pathways that contribute to harmful health effects resulting from a foodborne pathogen [39]. The numerical outcomes derived from this research’s *Salmonella* infection risk assessment should not be considered absolute. However, they highlight that ingesting salad vegetables, mainly when presented loosely and stored without refrigeration under unsanitary display conditions, is a risk factor for *Salmonella* infections in the UAE. It is essential to realize that the model estimate of the number of *Salmonella* infections per year for the entire UAE population does not indicate the number of disease cases; not all foodborne *Salmonella* infections result in disease, but some do. The symptoms of salmonellosis, when they do appear, can vary from mild to severe and include diarrhea, fever, and abdominal cramps. In severe cases, infections can result in serious complications such as meningitis or sepsis, especially in vulnerable populations [12]. It is essential to recognize that even if an individual does not manifest the symptoms of a *Salmonella* infection, they can still transmit the bacteria to others, hence why proper food and hand hygiene are critical in preventing the spread of infection [39].

The exposure estimation relied on several assumptions and simplifications for the risk assessment model used in this study, which is an inherent matter for any risk assessment model [39]. The model suffered from some limitations associated with the low detection rate, despite being based on a reasonable number of baseline data (n = 400). Five samples were *Salmonella*-positive in the present study; the confidence intervals cover the average prevalence of 1.25% for the combined tested items. Given the significant uncertainties per type of salad vegetable, it was impossible to distinguish the relative risks across the different types of vegetables. Secondly, the minimal *Salmonella* count data, substituted as the limit of detection, and such simplification might lead to an underestimation of the risk. This approach was acquired in our risk assessment based on a Dutch study evaluating infection risk with *Campylobacter* from consuming fruit and vegetables, where products detected as positive after selective enrichment were assumed to contain one CFU per 25 g, which might be higher [15]. Thirdly, the beta-Poisson dose–response formula was used to estimate the probability of infection with *Salmonella*. This formula does not account for differences in the pathogenicity of serovars found in the present study (e.g., Meleagridis, Mbandaka, and Anatum), given that the model parameters are based on studies focusing on selected serovars such as Enteritidis and Typhimurium [4]. Despite these limitations, this study represents the first attempt to use a quantitative risk assessment approach to guide risk managers on the potential contribution of leafy greens as a risk factor for salmonellosis in the UAE and throughout the Middle East. The risk assessment process is dynamic; thus, the utilized data, assumptions, and simulation may be updated as far as additional information, such as a greater amount of prevalence and concentration data or updated knowledge about consumer handling practices (e.g., temperature and washing practices), becomes available [4].

## 4. Conclusions

Our findings reveal that fresh salad vegetables sold in the UAE are generally safe for consumption regarding *Salmonella* occurrence, but occasional contamination is possible. Therefore, it is recommended that producers, retailers, and consumers should follow good hygiene practices, particularly for raw food items. By utilizing WGS, we conducted a comprehensive analysis of the genotypic characteristics of *Salmonella* retrieved from salad vegetables in the UAE, which involved detecting antimicrobial resistance, virulence determinants, and serotypes. The study isolates’ phenotypic and genotypic analysis suggests that fresh salad vegetables may offer limited exposure to antimicrobial-resistant *Salmonella* in the UAE. The calculated prevalence of *Salmonella* indicates that eating raw leafy greens should not be underestimated as a potential risk factor for *Salmonella* infections in the UAE. Unpackaged leafy vegetables such as arugula, spinach, and dill, presented in unrefrigerated conditions, pose a noteworthy risk. Given the popularity of such items in our daily meals, the number of *Salmonella* infections resulting from their consumption may continue to rise. The results of this study may be used for the future development of risk-based food safety surveillance systems and to elaborate on the importance of strengthening hygienic practices pre- and post-harvest.

## Figures and Tables

**Table 1:** The overall occurrence of *Salmonella* in salad vegetables sampled at retail in the United Arab Emirates.

*Salmonella* Prevalence				
No. of samples	400			
Number of samples positive for *Salmonella* (prevalence (P), %)	5 (1.25)			
* 95% Confidence interval of P	0.41–2.89			
Characterization of *Salmonella*-positive salad vegetable samples				
Sample code-type (sampling site)	Serovar	Sequence type	Antimicrobial resistance genes	Antimicrobial resistance phenotype
41–Arugula (supermarket, Al Ain)	28:z4,z24:z6	Novel	*aac(6’)-Ia* *a*	** Pan-susceptible
132–Dill (supermarket, Al Ain)	Meleagridis	463	*aac(6’)-Iaa, fosA7*	Pan-susceptible
278–Arugula (vegetable market, Dubai)	Mbandaka	3760	*aac(6’)-Iaa*	Pan-susceptible
279–Arugula (vegetable market, Dubai)	Mbandaka	3760	*aac(6’)-Iaa*	Pan-susceptible
335–Spinach (vegetable market, Dubai)	Anatum	64	*aac(6’)-Iaa*	Pan-susceptible

* The confidence intervals are calculated in STATA using the binomial distribution; ** refer to isolates showing non-susceptibility to all of the twelve antimicrobial agents in the study panel.

**Table 2:** Model parameters and values included in the risk assessment of *Salmonella* exposure from the consumption of salad vegetables in the United Arab Emirates.

Variable	Description	Units	Distribution/Calculation
*P_ret_*	Prevalence of non-typhoidal *Salmonella* in salad vegetables at retail in the UAE	%	Beta-distribution (α1; α1); where α1 = positive samples +1; α2 = total tested samples–positive samples +1 [23] RiskBeta (101; 87)–based on current study findings (Table 1)
*C*	The minimal *Salmonella* concentration in contaminated samples	CFU/g	Poisson-distribution of the limit of detection; RiskPoisson (1 CFU/25 g)–based on [15]
*M_veg_*	The average consumption (per day) of salad vegetables per individual in the UAE	g	Pert-distribution (Minimum; Most likely; Maximum) [23] RiskPert (12 g; 24 g; 36 g)–The average consumption based on the Food Balance Sheet survey (for the year 2019) of the Emirate of Abu Dhabi report [22]
*D*	The average dose/amount of *Salmonella* consumed (ingested) per idividual per day through a serving of salad vegetables	CFU	RiskPoisson (multiplication of: *P_ret_* × *M_veg_* × *C)*
*P_inf,day_*	The probability of infection with *Salmonella* per serving per day	Probability	RiskOutput (beta-Poisson dose–response formula with parameters alpha = 0.1324 (95% CI, 0.094 to 0.1817) and beta= 51.45 (95% CI, 43.75 to 56.39)–based on WHO/FAO [24]
*P_inf,year_*	The probability of infection with *Salmonella* per serving per year	Probability	RiskOutput (Calculation = *P_inf,day_* × 365)–based on [15]
*Cs_UAE,year_*	The expected number of cases of infection per year for the whole UAE population	Cases	RiskOutput (Calculation = *P_inf,year_* × number of UAE inhabitants (10.2 × 10^6^, based on Worldometer data [25]

**Table 3:** Outputs of the model depicting the infection risk among the United Arab Emirates inhabitants from the consumption of *Salmonella*-contaminated salad vegetables.

Simulated Model Output	Mean	90% Confidence Interval
Exposure (dose) of *Salmonella* ingested per individual per day via a serving of salad vegetables (*D*).	0.0131	±0.000524
The probability of infection with *Salmonella* per serving per day (*P_inf,day_*).	2.367 × 10^−5^	±1.320 × 10^−6^
The expected number of cases of infection over a year for the whole UAE population (*Cs_UAE,year_*).	10,584	±543

## Data Availability

The data presented in this study are available on request from the corresponding author.
